# Implementing an Oxygen Supplementation and Monitoring Protocol on Inpatient Pediatric Bronchiolitis: An Exercise in Deimplementation

**DOI:** 10.1155/2017/3169098

**Published:** 2017-10-18

**Authors:** Brian LeCleir, Leslie Jurecko, Alan T. Davis, Nicholas J. Andersen, Dominic Sanfilippo, Surender Rajasekaran, Anthony Olivero

**Affiliations:** ^1^Forest Hills Pediatrics, Grand Rapids, MI, USA; ^2^Quality and Safety Department, Helen DeVos Children's Hospital, Grand Rapids, MI, USA; ^3^Department of Surgery, Michigan State University, Lansing, MI, USA; ^4^Grand Rapids Medical Education Partners, Grand Rapids, MI, USA; ^5^Office of Research Administration, Spectrum Health, Grand Rapids, MI, USA; ^6^Department of Pediatric Critical Care Medicine, Helen DeVos Children's Hospital, Grand Rapids, MI, USA; ^7^Department of Pediatrics, Michigan State University College of Human Medicine, Grand Rapids, MI, USA

## Abstract

**Aim:**

Our goal in this study is to evaluate the effectiveness of our oxygen (O_2_) protocol to reduce length of stay (LOS) for children hospitalized with bronchiolitis.

**Methods:**

In this retrospective cohort study, the outcomes of children ≤ 24 months old that were admitted with bronchiolitis and placed on the O_2_ protocol were compared to historical controls. The primary outcome was hospital length of stay. Secondary outcomes were duration of O_2_ supplementation, rates of pediatric intensive care unit transfer, and readmission.

**Results:**

Groups were not significantly different in age, gender, and rates of respiratory distress score assessment. Significantly more severely ill patients were in the O_2_ protocol group. There were no significant differences between control and O_2_ protocol groups with regard to mean LOS, rates of pediatric intensive care unit transfer, or seven-day readmission rates. By multiple regression analysis, the use of the O_2_ protocol was associated with a nearly 20% significant decrease in the length of hospitalization (*p* = 0.030).

**Conclusion:**

Use of O_2_ supplementation protocol increased LOS in the more ill patients with bronchiolitis but decreased overall LOS by having a profound effect on patients with mild bronchiolitis.

## 1. Introduction

Lower respiratory tract infections such as bronchiolitis and viral pneumonia place tremendous strain on the health of young children and the healthcare system. The propensity of these viral infections to affect the most vulnerable of pediatric populations along with their highly variable clinical course leads to frequent hospital admissions that often occur regardless of severity [1]. The respiratory syncytial virus (RSV) alone affects roughly 800,000 children in the United States leading to approximately 20% of the annual birth cohort requiring medical attention yearly [2]. This results in $500 million of direct hospital costs in the United States alone [3].

Hypoxemia requiring supplemental oxygen (O_2_) is a key determinant in the decision to hospitalize infants with bronchiolitis and contributes to increased length of stay (LOS) [4]. Updated clinical practice guidelines released by the American Academy of Pediatrics (AAP) in 2014 identify O_2_ supplementation and hydration as the mainstay of treatment for bronchiolitis [5]. They establish a blood O_2_ saturation (spO_2_) of <90%, measured by pulse oximetry as a threshold for initiating O_2_ therapy and encourage discontinuing O_2_ supplementation and spO_2_ monitoring after improvement [6]. However, the integration of those guidelines can be quite challenging as they require ongoing “deimplementation,” a term describing the practice of discouraging care not supported by evidence-based research [7, 8]. These practices are difficult to discourage and often need a concerted effort to eliminate, even in the face of well-executed collaboration [9]. While there are numerous studies evaluating the impact of deimplementing interventions such as chest X-rays, antibiotics, and corticosteroids [10–16], few studies specifically evaluate the impact of a protocol based O_2_ supplementation practice [17–19]. Our institution developed and implemented an inpatient pediatric O_2_ supplementation and pulse oximetry protocol based on AAP guidelines [5] that aligns with the realities of our clinical practice. We hypothesize that the implementation of this protocol and deimplementation of certain practices lead to a significant decrease in hospital LOS for children admitted with bronchiolitis.

## 2. Methods

### 2.1. Intervention

**Figure 1:**
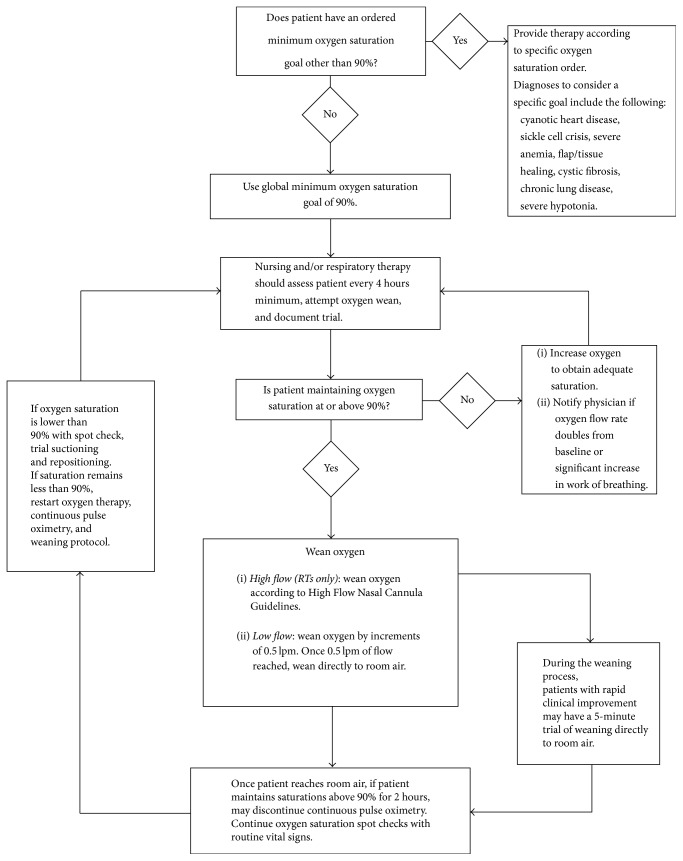
Standardized O_2_ supplementation and continuous pulse oximetry protocol.

A multidisciplinary team of clinicians at Helen DeVos Children's Hospital, a tertiary care pediatric hospital, designed and implemented a standardized O_2_ supplementation and continuous pulse oximetry protocol (Figure 1) in February 2013. Significant changes to prior medical practice included lowering the threshold for O_2_ supplementation from 94% to 90% and clearly defining both the steps and duration over which O_2_ supplementation would be titrated and discontinued. This protocol created a formal algorithm that was in clear contrast to a previously highly variable system that was driven by individual care providers. We performed education in the form of online modules, didactics, and formalized multidisciplinary rounds with respiratory therapists, nurses, and providers at the hospital. We also used education to stress and deemphasize some of the practices that were not supported by clinical evidence (Table 1).

### 2.2. Study Sample

In order to assess this quality initiative, we performed a retrospective review of the medical charts of infants and children (≤24 months of age) hospitalized with bronchiolitis during the preintervention study period of November 1, 2011, through April 30, 2012 (control group), and during the postintervention study period of November 1, 2013, through April 30, 2014 (O_2_ protocol group). Exclusion criteria included hospital admission directly to the pediatric intensive care unit (PICU), home O_2_ used immediately before or after hospitalization, presence of a tracheostomy tube, congenital heart disease, sickle cell disease, severe anemia, hypotonia, cystic fibrosis, and age > 24 months.

### 2.3. IRB Statement

Spectrum Health Institutional Review Board reviewed the study as a quality improvement (QI) project and thus was exempted from full review.

### 2.4. Data Sources

The medical records of patients who met the entry criteria with discharge diagnoses of “acute bronchiolitis” were reviewed. Patients were identified using electronic medical record search queries for International Classification of Diseases, Ninth Revision (ICD-9), primary or secondary diagnosis codes 466.1 (acute bronchiolitis), 466.11 (acute bronchiolitis due to respiratory syncytial virus), and 466.19 (acute bronchiolitis due to other infectious organisms).

### 2.5. Study Variables

Patient age at admission, gender, respiratory distress score (RDS), duration of supplemental O_2_, LOS, seven-day readmission rate, rate of PICU transfer, and coexisting medical problems were collected. LOS, as noted in hours, was determined as the period from the time of admission to the inpatient unit until the time of discharge. We evaluated bronchiolitis illness severity at the time of the first respiratory therapist evaluation by using a modified RDS tool, an evaluation tool based on respiratory rate, accessory muscle use, wheezing, O_2_ requirement, and inspiratory to expiratory ratio (Table 2) [20]. RDS values ≥3 were considered to be indicative of moderate to severe bronchiolitis.

### 2.6. Statistical Methods

The data was analyzed using IBM Statistics SPSS v. 21 (Armonk, New York). Quantitative data was compared using a *t*-test and was reported as the mean ± SD. Nominal data was compared using the *χ*^2^ test and Fisher's exact test (when appropriate) and was reported as percentages. Due to the nonnormal distribution of LOS and duration of O_2_ supplementation, both of these variables were transformed prior to analysis, while the summary statistics shown are for the untransformed data. The LOS was transformed using the natural log, while the duration of O_2_ supplementation was transformed using the inverse hyperbolic sine. In addition, a multiple regression analysis was performed, using the log transformed LOS as the dependent variable, with patient age, O_2_ protocol group versus control group, PICU transfer, and RDS score as the independent variables. Significance was assessed at *p* < 0.05.

## 3. Results

From the collective study periods, a total of 263 children met the study criteria: 141 children in the control group and 122 children in the O_2_ protocol group.  Table 3 shows the patient's demographics and characteristics. There were no significant differences based on age, gender, and PICU transfer rates between control and O_2_ protocol groups (Table 3). Only three children were readmitted within seven days, one in the control group and two in the O_2_ protocol group.

Contrary to our hypothesis, there was not a statistically significant difference in LOS between the control and O_2_ protocol groups. However, the O_2_ protocol group had a significantly higher severity of illness at admission based on their initial RDS independent of assessment rates.

Next, we assessed the relationship between LOS and bronchiolitis severity, as defined by the RDS. Patients in the O_2_ protocol group with mild bronchiolitis (RDS < 3) had a statistically significant shorter LOS compared to control (RDS ≥ 3) (*p* = 0.005). Interestingly, O_2_ protocol group subjects with moderate to severe bronchiolitis (RDS ≥ 3) had a 29% increase in LOS, although this was not statistically significant (*p* = 0.535). Furthermore, O_2_ protocol group subjects with mild disease had a significantly shorter LOS compared with the O_2_ protocol patients with moderate to severe bronchiolitis (Table 4). This data suggests that LOS may be dependent on disease severity. O_2_ protocol group subjects with mild bronchiolitis disproportionately improve compared with moderate to severe disease. Furthermore, the O_2_ protocol may have a negative affect when bronchiolitis is more severe. However, no direct conclusive relationship between LOS and the protocol could be inferred for moderate to severe disease due to the disproportionately higher RDS patients in the O_2_ protocol.

We performed a multiple regression analysis to independently assess each subject group and variables affecting the LOS (Table 5). We found age, RDS, and PICU transfer all had a significant correlation with LOS. A one-month increase in age significantly decreased LOS. RDS significantly impacted LOS, whereas a one-unit increase in RDS significantly increased LOS by 11.3% and a two-unit increase in RDS increased LOS by 23.9%. Transfer to the PICU significantly increased LOS by 2.9 fold. The O_2_ protocol had a significant inverse association with LOS compared to the control group; patients in the O_2_ protocol group had a 19.7%, decrease in LOS (Table 4).

## 4. Discussion

High variability in clinical care often contributes to higher healthcare costs and poor adherence to evidence-based practices [21]. For this reason, health care professionals have developed protocols to drive therapies and reduce the lack of concordance. Studies have shown clinical outcomes from nonphysician directed protocols compare favorably with physician driven interventions in multiple settings [22–24]. Such protocols used in the PICU have the potential to save money and reduce resource allocation when used in the non-ICU setting. Our study suggests that this is potentially true. We reduced LOS for patients with mild bronchiolitis (initial RDS < 3) after the implementation of the O_2_ protocol and deimplementation of unnecessary practices. However, LOS appeared to increase for patients with higher RDS following protocol implementation. The actual impact of RDS on LOS is somewhat confounded by the fact that RDS was higher in the postimplementation years even though the rates of assessment were similar in both groups. When we controlled for the RDS effect by using multivariate regression analysis there was a demonstrable aggregate benefit that was more than compensated for the increased LOS in sicker patients.

Deimplementation and deinnovation are quality improvement (QI) terms that emphasize the abandonment of unnecessary care that is not supported by evidence-based research [7, 8]. These terms focus on the ideal of discouraging use rather than discouraging underuse and have been used in the context of eliminating nonevidenced-based practices in bronchiolitis [9]. We paired the initiation of O_2_ use protocol along with deimplementation of unnecessary practices and hypothesized that a collective approach would have specific value in reducing unnecessary care. This provided a mechanism to overcome the inertia of so-called “established” clinical practice and increase the provider's sense of efficacy.

The AAP prioritizes the prevention of unnecessary care [5, 6], and a recent study showed benefit to eliminating practices such as X-rays and alpha-agonist therapy in community hospital settings [9]. The deimplementation of such practices requires constant education. It was our experience that the respiratory therapists were the strongest advocates for avoiding such unnecessary therapy. Applications of nebulized *β*-agonist therapy and/or hypertonic saline were rarely tried, and when attempted, they were discontinued once lack of efficacy was established. Such an approach has benefit in saving money, eliminating unnecessary interventions, and focusing care on the sickest during the time of the year that hospitals are busiest [25].

There is wide variation in the clinical course of bronchiolitis and thus it is difficult to distinguish which patients will require only titration of O_2_ therapy from those who will require a more involved escalation of care [26, 27]. For example, there have been efforts in the past to define clinical criteria that could predict inpatient LOS for children with bronchiolitis that have shown that initial spO_2_ values do not predict LOS in children with bronchiolitis [26]. Our protocol attempts to surmount some of that dilemma by predicating appropriate care and letting the clinical course dictate the level of care the patient receives.

This study does have limitations in that the single center retrospective design with historical controls limits our ability to conclusively state that there is benefit to the O_2_ supplementation protocol. This study design by nature is an observational study. It is possible that natural cycle of viral virulence, unknown changes to childhood immunity, and other unknown variations may affect this study. In addition, other interventions independent of this oxygen protocol may have played a role in these outcomes, in particular LOS. One criticism for our study might be that the RDS score we used is more of an amalgam of other scores available in the literature rather than a validated score. However, the score was uniformly applied to all patients, and clearly an increase in the score was associated with an increase in respiratory distress. Also, the RDS scores were notably higher on average in the O_2_ protocol group. It is possible this is due to a true difference in illness severity between the seasons evaluated, or the increased frequency of respiratory therapist monitoring mandated by the O_2_ protocol resulted in increased provider confidence in keeping patients with higher illness severity on general inpatient floors. Regardless, we presented data that the weaning protocol might have benefit in a selected group of milder bronchiolitis cases.

## 5. Conclusion

This study is unique in that it highlights both the benefit and the unforeseen effect of applying protocols to patient care. Our application of the AAP guidelines in a collaborative manner saw a decrease in LOS of children with milder bronchiolitis while LOS for the sicker patients increased. However, the overall effect was one of the benefits with potential to exert an impact on appropriate hospital triage and cost. This translates into a benefit for both patients and their families.

## Figures and Tables

**Table 1:** Clinical practices discouraged on initiation of protocol.

Discouraged	Encouraged
Chest radiography	Weaning O_2_ flow and FIO_2_
Viral panel testing	Accepting saturation of >90% if child does not appear distressed
Blood draws for laboratory testing	Discontinuing supplemental gas flow when patient is on room air
*β*-Agonist therapy	Calling the physician for any perception of deterioration
Steroids	Spot checks in pulse oximetry
Antibiotic therapy	

**Table 2:** Respiratory distress score (RDS) calculation.

Respiratory distress score
Respiratory rate
□ 0 Normal (respiratory rate (rr) up to 40 breathes/minute)
□ 1 Elevated (RR 40–60 breaths/minute)
□ 2 Tachypnea (RR greater than 60 breaths/minute)
Accessory muscle use
□ 0 Normal
□ 1 Retractions (substernal, subcostal, intercostal)
□ 2 Neck or abdominal muscle use
Wheezing
□ 0 None or scattered end expiratory wheezes
□ 1 Wheezes throughout expiration
□ 2 Entire inspiration and expiration wheezes
Oxygen requirement
□ 0 Maintains SpO_2_ above 90% on room air
□ 1 Maintains SpO_2_ above 90% on less than 1 Lpm oxygen
□ 2 Requires 1 Lpm oxygen or more to maintain SpO_2_ above 90%
Inspiratory to expiratory ratio
□ 0 I : E ratio less than 1 : 2
□ 1 I : E ratio 1 : 2 to 1 : 3
□ 2 I : E ratio greater than 1 : 3

**Table 3:** Demographic and clinical variables.

Variable	Control group (*n* = 141)	O_2_ protocol group (*n* = 122)	*p* value
Age (months)	6.2 ± 5.5	7.0 ± 6.3	0.31
Gender: male/female	77 (54.6%)/64 (45.4%)	70 (57.4%)/52 (42.6%)	0.65
RDS assessed	125/141 (88.7%)	110/122 (90.2%)	0.69
RDS	2.0 ± 1.5	2.7 ± 1.6	<0.001
LOS (h)^a^	69.6 ± 67.5	72.5 ± 77.4	0.374
LOS: RDS < 3^a,b^	70.6 ± 60.3	51.6 ± 42.6	0.005
LOS: RDS ≥ 3^a,c^	74.0 ± 53.4	95.2 ± 95.7	0.535
Duration of O_2_ supplementation (h)^a^	38.3 ± 58.4	40.9 ± 62.9	0.638
Number requiring supplemental O_2_	134/141 (95.0%)	113/122 (92.6%)	0.414
PICU transfer	10/141 (6.7%)	13/122 (9.7%)	0.310
7-day readmission	1/141 (0.7%)	2/122 (1.6%)	0.598

Data are presented as the mean ± SD or as percentages; O_2_, oxygen; LOS, length of stay; RDS, respiratory distress score; PICU, pediatric intensive care unit. ^a^Data were analyzed using log transformed data, values shown are untransformed data. ^b^Control group *n* = 87; oxygen protocol group *n* = 49. ^c^Control group *n* = 38; oxygen protocol group *n* = 61.

**Table 4:** Study group LOS^a^ and PICU transfer compared by RDS.

Outcome measure	RDS < 3 (*n* = 136)	RDS ≥ 3 (*n* = 99)	*p* value
Control group LOS (h)^b^	70.6 ± 60.3	74.0 ± 53.4	0.697
O_2_ protocol group LOS (h)^c^	51.3 ± 41.4	92.4 ± 94.3	0.005
PICU transfer	9/136 (6.6%)	12/99 (12.1%)	0.144

Data are presented as the mean ± SD or as percentages; O_2_, oxygen; LOS, length of stay; RDS, respiratory distress score; PICU, pediatric intensive care unit. ^a^Data analyzed using log transformed data, values shown are untransformed data. ^b^RDS < 3 group *n* = 87; RDS ≥ 3 group *n* = 38; ^c^RDS < 3 group *n* = 49; RDS ≥ 3 group *n* = 61.

**Table 5:** Multiple regression analysis, with log transformed length of stay (LOS) as the dependent variable.

Variable	*β*-Coefficient	95% CI	*p* value
O_2_ protocol group^*∗*^	−0.22	−0.42–−0.02	0.030
Age	−0.02	−0.04–−0.01	0.008
RDS	0.11	0.04–0.17	0.001
PICU Transfer	1.06	0.72–1.41	<0.001

CI, confidence interval; O_2_, oxygen; RDS, respiratory distress score; PICU, pediatric intensive care unit. ^*∗*^Control group (reference group) versus the O_2_ protocol group.

## Abbreviations

RSV: Respiratory syncytial virus
O_2_: Oxygen
LOS: Length of stay
AAP: American Academy of Pediatrics
spO_2_: Blood oxygen saturation
PICU: Pediatric intensive care unit
QI: Quality improvement
ICD-9: International Classification of Diseases, Ninth Revision
RDS: Respiratory distress score.
